# Editorial: Advanced green and sustainable chemical and physical technologies for resources recycling of solid wastes

**DOI:** 10.3389/fchem.2023.1146404

**Published:** 2023-01-24

**Authors:** Xiangning Bu, Ilhwan Park, Ugur Ulusoy

**Affiliations:** ^1^ School of Chemical Engineering and Technology, China University of Mining and Technology, Xuzhou, China; ^2^ Division of Sustainable Resources Engineering, Faculty of Engineering, Hokkaido University, Sapporo, Japan; ^3^ Department of Chemical Engineering, Cumhuriyet University, Sivas, Türkiye

**Keywords:** sustainable mining, e-waste, mineral waste processing, metals recycling, solution purification, zero-waste recycling, acid leaching, metal extraction

To prevent CO_2_-induced climate change, the world is quickly moving toward a carbon-neutral society by using electric vehicles, renewable energy sources, and other energy sources, which demand more resources than traditional ones in terms of materials, minerals, and metals. In this regard, recycling processes of rare earth elements (REEs), metals, plastic, and glass from secondary sources with a zero-waste strategy have become more important in order to reduce environmental damage and bring them into the economy when primary mineral resources are running out. Therefore, in this Research Topic, studies on effective, non-hazardous, long-term, and ecological recycling processes of solid wastes, including by-products from industrial processes containing metals have been compiled.

This Research Topic, which selects and collects eleven original research papers and one mini-review paper that has conducted studies on this field, sheds fresh light on critical aspects providing crucial scientific knowledge that will benefit future research. The first part of the articles collected is related to the release and migration of heavy metals in solid wastes. Zhang et al. calculated the environmental vulnerability factors affecting the dissolution of heavy metals in fly ash from a thermal power plant. Fly ash has been suggested as a possible soil conditioner and additive that might be used to enhance reclamation soil in coal mining subsidence sites. Zheng et al. analyzed how low molecular weight organic acids such as citric acid and malic acid impacted the migratory characteristics of Pb in polluted soils. Due to its capacity to activate Pb, the use of citric acid has been suggested as a technique that can improve the efficacy of remediating reclaimed soil. Since it has been reported that the smelting technique has the potential for innocuous processing to overcome the waste Research Topic in the electrolytic manganese industry by creating glass ceramics using electrolytic manganese slag as feedstock and solidified heavy metal constituents (Sun et al., 2020), Wang et al. studied the process of heavy metal solidification and stability in glass-ceramics containing electrolytic manganese slag. It has been concluded that the glass-ceramic system’s interwoven pattern of glass and crystal phases also contributed to the improved curative influence of heavy metals. In addition, Hao et al. explored the possibility of making glass ceramics from wastes such as coal gasification slag and petrochemical incineration fly ash (PIFA) by trapping dangerous toxic metals in their crystalline-noncrystalline multistage composition.

The second part of the collected papers are on resource recycling of solid wastes using chemical and physical separation techniques. Phann et al. demonstrated that Alanine is an ecologically friendly option for the preferential leaching of used hydrodesulfurization catalysts containing Mo and Co metals. Huang et al. introduced ultrasonication to enhance the oxidation leaching of chromium from electroplating sludge. In addition to leaching, Li et al. proposed innovative, inexpensive, and hugely beneficial recycling of carbon fiber-reinforced polymer (CFRP) wastes using the swelling agent of dimethylacetamide (DMAC) for diminishing the loss of carbon fiber length and strength. Wang et al. investigated the recovery of discarded carbon anode slag from aluminum electrolysis by flotation. It was found that carbon anode slag demonstrated a higher flotation selectivity under the optimized grinding flotation compared to direct flotation. For advanced physical separation, Phengsaart et al. designed a new technique named as the reverse hybrid jig integrating the methods of jigging and flotation to separate plastics (polypropylene/polyethylene) and suggested a new index using concentration criterion for predicting the effectiveness of the separation.

The rest of the collected papers are related to the preparation of the brazing alloy using recycled E-waste and arsenic removal in gold-smelting wastewater by the modified agricultural mulch film residual. Since the majority of the metals used in circuit boards can serve as parts of the multi-element alloy brazing alloy, Yang et al. have utilized discarded mobile phones for e-waste recycling by investigating the contents, smelting point, form, and characteristics as well as the size, form, and other microstructure development of the second phase of brazing alloy created by smelting the circuit boards. The work of Zhang et al. proposed an innovative and efficient method to purify and remove arsenic from gold-smelting wastewater by utilizing agricultural mulch film residual followed by an iron modification to avoid the harmful effect of arsenic on humans and the environment.
